# Evaluating the validity of the Amharic Brief Pain Inventory among people with chronic primary musculoskeletal pain in Ethiopia

**DOI:** 10.1186/s12891-022-05833-5

**Published:** 2022-09-21

**Authors:** Abey Bekele Abebe, Tadesse Awoke Ayele, Jordan Miller

**Affiliations:** 1 School of Rehabilitation Therapy, Queen’s University, Kingston, ON Canada; 2 Department of Physiotherapy, College of Medicine and Health Sciences, University of Gondar, Gondar, Ethiopia; 3 Department of Epidemiology and Biostatistics, Institute of Public Health, College of Medicine and Health Sciences, University of Gondar, Gondar, Ethiopia

**Keywords:** Chronic primary musculoskeletal pain, Cross-cultural validation, Confirmatory factor analysis, Ethiopia

## Abstract

**Background:**

The Brief Pain Inventory (BPI) is a multidimensional pain assessment tool used to evaluate pain severity and pain interference. The BPI has been translated and validity estimated across multiple languages and patient populations for clinical and research settings. This study aimed to assess the reliability and validity of Amharic BPI test scores among patients with chronic primary musculoskeletal pain living in Ethiopia.

**Methods:**

This study had two parts: cognitive interviews and psychometric testing. An expert committee reviewed the Amharic BPI, and fifteen participants participated in the cognitive interviews. The results from the cognitive interviews were evaluated, and the committee approved recommendations for the tool prior to psychometric testing. Two hundred and sixty-nine patients were recruited from three sites for the psychometric testing. The results were summarised using descriptive statistics. Cronbach’s alpha was calculated to estimate the internal consistency. To assess test-retest reliability, the intraclass coefficient was examined, and a Bland-Altman plot was created. Construct validity was determined using confirmatory factor analysis by testing BPI’s previously suggested two or three-factor dimensionalities. Convergent validity was assessed by estimating the correlation between the Amharic BPI and SF-36 subscales.

**Results:**

The Amharic BPI scores showed a good internal consistency using a 2-factor model with α = 0.89 for pain severity and α = 0.91 for pain interference. Good internal consistency was also observed in the 3-factor model, with α = 0.89 for pain severity, α = 0.84 for activity interference, and α = 0.86 for affective interference items. The test-retest reliability testing resulted in an ICC = 0.82 for pain severity and ICC = 0.90 for the pain interference. The severity scale had the highest correlation with bodily pain subscale of the SF-36 at *r* = − 0.44, and the interference scale with Physical functioning scale of SF-36 at *r* = − 0.63. Confirmatory factor analysis support rating Amharic BPI using a two-factor approach.

**Conclusions:**

Our findings showed that Amharic BPI scores demonstrate internal consistency, test-retest reliability, and construct validity among patients with chronic primary musculoskeletal pain in Ethiopia. Accordingly, the tool can be used in clinical practice or research in similar settings.

## Background

Chronic pain is a prevalent, complex, and debilitating condition that has far-reaching consequences on individuals, society, and the healthcare system [1, 2]. Chronic pain prevalence estimates range from 20 to 25%, depending on the country or region [3]. Around 1.71 billion individuals suffer from musculoskeletal pain disorders, specifically low back pain accounting for most of the burden [1]. An increasing prevalence of musculoskeletal pain in Ethiopia has been reported in recent studies [4, 5]. Research on chronic musculoskeletal pain in Ethiopia has been quite limited. This may be partly due to the lack of assessment tools validated for use in the Ethiopian context.

Pain assessment in clinical or research contexts should consider different factors to encompass the complex nature of pain. Pain assessment includes gathering information on the nature, duration, body location, progression of pain, and effects on daily activities [6]. Multimodal pain assessment techniques assess the level of pain intensity and pain effect on the person’s daily physical, mental, and social functions [7, 8]. Creating or adapting pain assessment tools that provide valid and reliable measures of multiple dimensions of pain is critical to assessing chronic pain in clinical practice and research in Ethiopia.

The Brief pain inventory (BPI) is a multidimensional self-administered pain assessment tool. The BPI has two dimensions assessing pain intensity and pain interference on the patient’s Activities Of Daily Living (ADLs) [9]. It was first developed in English by Cleeland [10] and validated for use among cancer pain patients. Since then, the BPI has been translated into numerous languages worldwide, and its validity has been tested to assess patients with a variety of health conditions. For example, BPI was translated to German [11], Hindi [12], Norwegian [13], and Amharic [14] to assess cancer pain, to Thai to assess postoperative cardiac surgery pain [15], to Persian to assess chronic pain [16], and to Turkish to assess musculoskeletal pain [17].

Internal consistency estimated different versions using Cronbach’s alpha ranged from 0.80–0.87 for pain severity scale and from 0.89–0.92 for pain interference scales [10, 14, 17, 18]. Previous studies have also shown a good test-retest reliability of Intra-class coefficient (ICC) ranging from 0.79–0.92 for the pain severity scale and an ICC ranging from 0.81–0.93 for the pain interference scale [9, 10].

BPI has been translated into Amharic, and its validity has been estimated among patients with cancer pain living in Ethiopia [14]. The results suggest three factors for Amharic BPI: pain severity, physical activity interference, and psychosocial interference with the internal consistency of Cronbach’s α coefficients of 0.85, 0.87, and 0.77, respectively. Furthermore, the pain severity and pain interference scores had intraclass correlation values of 0.75 and 0.78, respectively, for test-retest reliability [14].

The validity of Amharic BPI has not been assessed for use among patients with chronic primary musculoskeletal pain, which represents a substantial proportion of the population with pain globally [19] and in Ethiopia [4, 5, 20]. Given the unique experiences of people with chronic primary musculoskeletal pain compared to those with cancer-related pain, estimating the reliability and validity of Amharic BPI in this patient group is essential prior to use in clinical practice and research. This study aimed to assess the reliability and validity of the Brief Pain inventory Amharic test scores among patients with chronic primary musculoskeletal pain living in Ethiopia using cognitive interviews and psychometric testing.

## Methods

### Aim

The aim of this study was to assess the reliability and validity of the Amharic Brief Pain Inventory test scores among patients living in Ethiopia with chronic primary musculoskeletal pain using cognitive interviews and psychometric testing.

### Design

This research was conducted in two phases. The first phase of the research was to conduct think-aloud cognitive interviews [21] to make sure the translated tools were adapted to the language and culture and considered appropriate for use by Ethiopian adults with chronic primary musculoskeletal pain. The second phase of the research was a cross-sectional study to test the construct validity, internal consistency, and test-retest reliability of the Amharic BPI scores among patients with chronic primary musculoskeletal pain living in Ethiopia.

### Setting and participants

#### Phase-1: cognitive interview

Fifteen adult participants [22] with chronic primary musculoskeletal pain were recruited from the Gondar University Referral Hospital to participate in the cognitive interviewing. Purposive sampling was used to ensure representation of both men and women, people with pain in multiple body regions, and people who live in urban and rural areas.

#### Phase-2: psychometric testing

We aimed to recruit a minimum of 253 participants based on the BPI having 11 items, using a 20:1 respondent-to-item ratio [23], and allowing for up to a 15% dropout rate for the test-retest reliability estimates.

Participants included were: 1) patients with chronic primary musculoskeletal pain [24]; 2) adults between the ages of 18 and 64 [25]; and 3) patient who would communicate in Amharic. Participants excluded were: 1) patients with chronic secondary pain or other chronic primary pain diagnoses (e.g. chronic widespread pain, complex regional syndrome, chronic primary headache or orofacial pain, and chronic primary visceral pain) [26], and; 2) patients who had chronic primary musculoskeletal pain but were being treated in the hospital for another reason.

For the purposes of this study, chronic primary musculoskeletal pain was defined using the 2019 International Association for the Study of Pain (IASP) classification system for chronic pain. In this classification, chronic musculoskeletal pain is defined as pain located in the muscles, bones, joints, or tendons [24]. Participants with pain in any body region were eligible (i.e., neck pain, back pain, upper extremity pain, lower extremity pain) as long as they met the definition of chronic musculoskeletal pain.

The participants for the psychometric testing were recruited from three research sites in Ethiopia: Gondar University Referral Hospital in Gondar, Felege Hiwot Referral Hospital in Bahir Dar, and Black Lion referral Hospital in Addis Ababa. These sites were selected as they were among the largest referral hospitals in Ethiopia. The study’s goal was to estimate the validity of the Amharic BPI for use with Amharic-speaking individuals anywhere in Ethiopia. Recruitment from these three sites allowed for a more diverse sample to align with our objectives.

### Measures

Brief pain inventory (BPI): BPI is a two-dimensional pain assessment tool designed to evaluate pain severity and pain interference over the previous 24 hours [27]. The pain severity scale has four items: pain at its “worst,” “least,” “average,” and “pain right now”. Each item is rated on a 0–10 scale (0 = no pain and 10 = pain as bad as you can imagine) and the mean of the four items is used to calculate a single pain severity index. The pain interference scale has seven items asking patients to rate the level of pain interference on a 0–10 scale (0 = do not interfere and 10 = completely interferes) on their general activity, mood, walking ability, normal work, relationship with others, sleep, and enjoyment in life [27]. The mean value of the seven items is calculated to provide a single pain interference index score [9, 27]. A higher score indicates more severe pain or greater pain interference. The BPI was previously translated to Amharic, and validity was estimated among patients with cancer-related pain [14]. We used this tool as a starting point for this study. Consent was obtained from the original tool developer Dr. Charles Cleeland (University of Texas M. D. Anderson Cancer Center, Houston, TX, USA) and the Amharic BPI translator Ephrem Engidawork Ph.D. (Department of Pharmacology and Clinical Pharmacy, School of Pharmacy, College of Health Sciences, Addis Ababa University, Ethiopia).

Short-form health survey (SF-36): SF-36 is a health-related quality of life measurement tool with 36 items that form 8 scales [28, 29]. These scales are 1) Physical functioning, 2) Role limitations due to physical health, 3) Role limitations due to emotional problems, 4) Energy/fatigue, 5) Emotional well-being, 6) Social functioning, 7) Pain, and 8) General health [30]. There are two steps in scoring SF-36. First, the pre-coded numeric scoring will be re-coded using the scoring key. Each item’s score will be re-coded on a scale of 0 to 100, with 0 representing the lowest possible score and 100 representing the highest possible score. All items are scored, with a higher score indicating a better health state. Second, the eight scale scores will be calculated by averaging the items on the same scale [29, 31]. The SF-36 has been translated into Amharic and validated among the Ethiopian Population [32].

### Data collection

#### Phase-1: cognitive interview

An expert committee was formed consisting of two clinicians, two language experts and the primary investigator. The committee reviewed the existing Amharic BPI and the original BPI English questionnaires and confirmed the appropriateness of the translation of the initial Amharic BPI before conducting the cognitive interviews. Demographic information was collected from participants using a paper-based survey and cognitive interviews were conducted by one interviewer (AA) using the Amharic BPI and a semi-structured interview guide. The cognitive interview participants were not included in the psychometric testing.

#### Phase-2 psychometric testing

Two trained data collectors collected the data for psychometric testing at each site. After obtaining informed consent, the data was collected using a patient demographics form, the BPI, and the SF-36. For the test-retest, the data was collected again for all measures between 3 days and 1 week after the initial data collection [33, 34]. The time between test and retest was chosen as a timeline associated with low likelihood of change in people with chronic musculoskeletal pain and allowing enough time between measures to reduce recall bias [33, 35]. It also had a pragmatic benefit of allowing us to include patients who came from outside the city where the hospital is located. Patients frequently stay in the city for several days to receive initial care from the referral hospital prior to returning to their rural home or community. Data was collected from November 2020 to April 2021.

### Analysis

#### Phase-1: cognitive interview

The cognitive interviews were analysed using cross-case item analysis [36]. The participants’ perspectives on comprehensibility, cognitive equivalence, cultural equivalence, and alternative translations for the individual items of the questionnaire were analysed [37]. The expert committee reviewed the results, discussed, and approved the final version for the psychometric testing.

#### Phase-2 psychometric testing

The collected data for the psychometric testing was compiled in SPSS version 27 Statistical software [38] for analysis. For quality assurance, 20% (*n* = 53) of the data was randomly cross-checked with the data on paper. The data set was then visually checked for missing data and entry errors, and further descriptive statistics were conducted for each variable to detect outliers [39, 40].

Descriptive statistics (mean, SD, frequency, and percentage) were used to report participants’ demographics and clinical information. The assumption of normality was tested using Q-Q plots and Shapiro-Wilk before conducting the reliability and validity tests [41]. Internal consistency was assessed using Cronbach’s alpha. Based on the previous studies [42], Cronbach’s alpha ≥0.70 was considered as an acceptable internal consistency value. In the case of test-retest reliability, the intraclass coefficient (ICC) was calculated, and ICC ≥ 0.70 was considered an acceptable test-retest reliability [43, 44].

Construct validity was estimated using confirmatory factor analysis by testing the previously suggested two or three-factor dimensionalities of the BPI [16, 18, 42, 45] using IBM SPSS Amos version 27 [46]. Chi-Square/ degree of freedom (X^2^/df), Comparative Fit Index (CFI), Tucker–Lewis’s index (TLI), Root mean Square Error of Approximation (RMSEA) (CI 90%), Standardized Root Mean Residual (SRMR), Adjusted Goodness of fit (AGFI) were calculated to determine the best fit model between the 2-factor and the 3-factor models.

A ratio of X^2^/df less than 5 [47] and values for CFI greater than 0.95, TLI greater than 0.95, RMSEA at or less than 0.08, SRMR less than 0.08 and AGFI greater than 0.8 indicate a good fit [48, 49].

Convergent validity was tested by calculating Pearson’s correlation coefficient with each of the subscales of the SF-36 [45]. Correlation coefficients of ≤0.29 were considered weak, 0.30–0.49 as low, 0.50–0.69 as moderate, and ≥ 0.70 was considered a strong correlation [50]. It was hypothesized that there would be a moderate-high correlation between the BPI pain severity index and the pain scale of the SF-36 and a moderate correlation between the BPI pain interference index with functioning scales of the SF-36 (See Table 1 below).

**Table 1:** Hypotheses for correlation between BPI and SF-36

^a^SF-36 scores	^b^BPI pain Severity (Mean Score)	^b^BPI pain Interference (mean score)
**Physical functioning**	Low-moderate negative correlation	Moderate negative correlation
**Role limitations due to physical health**	Low-moderate negative correlation	Moderate negative correlation
**Role limitations due to emotional problems**	Low-moderate negative correlation	Moderate negative correlation
**Energy/fatigue**	Low-moderate negative correlation	Moderate negative correlation
**Emotional well-being**	Low-moderate negative correlation	Moderate negative correlation
**Social Functioning**	Low-moderate negative correlation	Moderate negative correlation
**Bodily Pain**	Moderate-high negative correlation	Moderate negative correlation
**General Health**	Low-moderate negative correlation	Moderate negative correlation

^a^Sf = 36 scores: Higher scores indicate less impact on life, while lower scores indicate greater impact

^b^BPI scores: Higher scores indicate a greater impact on life, while lower scores indicate a lesser impact

## Results

### Cognitive interview results

Fifteen participants (9 Females and 6 Males) with chronic primary musculoskeletal pain were recruited for the cognitive interview. All participants were assessed by a physiotherapist at the University of Gondar physiotherapy clinic, who screened for eligibility. They were scheduled for a separate appointment for the cognitive interview. We interviewed participants with a range of demographics (age, sex, living area, and education status). As there is a high illiteracy rate in Ethiopia [51], participants were consistently provided with a paper copy of the BPI and also read each item aloud. Participants provided their responses verbally.

According to the cognitive interview responses, the BPI questions were clear. However, during the interview, we noticed that most participants paused for a second to comprehend ‘average pain,’ which was translated as  in Amharic. This translation gives ‘average’ a more mathematical meaning. Based on the participants’ responses and the committee discussion, the translation was changed to  , which gives ‘average pain’ a meaning that is equivalent to ‘typical pain’ or ‘common pain.’

### Psychometric test results

#### Patient characteristics

**Fig. 1:**
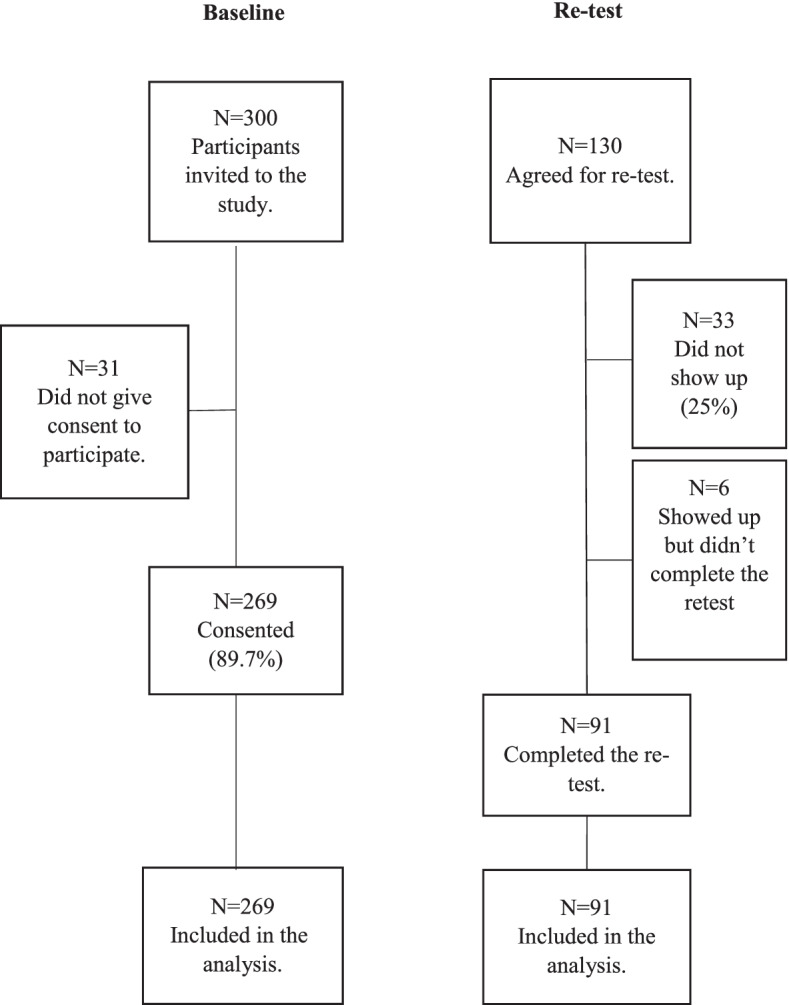
The study sample and data collection flowchart for assessing the psychometric properties of the Amharic BPI. The Bland-Altman plot for the test-retest reliability of the BPI pain severity scale. The difference of Test 1 and Test 2 is plotted against their mean, together with the mean of the difference, 95% Confidence interval (CI), and upper and lower limit of agreement (LOI)

We invited 300 people to take the Psychometric test, and 269 of them agreed. All 269 participants were included in the study. Only 91 of the total participants participated in the test-retest; the others did not agree to participate, did not show up, or showed up but did not complete the test-retest (See Fig. 1 below).

The mean age of the participants was 41.2 (SD = 12.4). The majority of the participants were female (145, 53.9%), resided in Gondar (139, 51.7%), and had completed college or university education (105, 39.0%). The mean BPI severity score was 3.96 (SD = 1.99), and the mean BPI interference score was 4.13 (SD = 2.27) (See Table 2 below).

**Table 2:** Demographic and clinical characteristics of participants

Characteristics	Aggregate
**Age – years (mean, SD)**	41.19 (12.4)
**Sex** (n, %)	
Male	124 (46.1)
Female	145 (53.9)
**Educational status** (n, %)	
None	20 (7.4)
Primary Level	70 (26.5)
Secondary Level	74 (27.5)
College or university education	105 (39.0)
**Employment status (n, %)**	
Unemployed	29 (10.8)
Employed	119 (44.2)
Private	77 (28.6)
Others	44 (16.4)
**Address (n, %)**	
Gondar	139 (51.7)
Addis Ababa	91 (33.8)
Bahir Dar	39 (14.5)
**First diagnosed By (n, %)**	
Physiotherapist	100 (37.2)
MD	163 (60.6)
Others	6 (2.2**)**
**Current interventions (n, %)**	
None	2 (0.7)
Physiotherapist	157 (58.4)
Medication	6 (2.2)
Physiotherapist and medication	104 (38.7)
**Area of pain (n, %)**	
Neck	25 (9.3)
Thoracic	24 (8.9)
Shoulder	30 (11.2)
Upper limb	15 (5.9)
Lower back	161 (56.9)
Pelvic	20 (7.4)
Lower limb	26 (9.7)
**Duration of Pain (in months) (mean, SD)**	26 (31.94)
**BPI pain severity score (mean, SD)**	3.96 (1.99)
**BPI pain interference score (mean, SD)**	4.13 (2.27)
**SF-36 (0–100)**	
**General health perception (GH)**	52.83 (18.72)
**Bodily Pain (BP)**	44.20 (22.01)
**Physical functioning (PF)**	51.15(23.25)
**Role Functioning Emotional (RLEP)**	20.20 (33.10)
**Energy (EN)**	56.450 (18.40)
**Emotional Well-Being EM)**	56.88 (18.73)
**Social Functioning (SF)**	60.33 (22.29)
**Role Functioning Physical (RLPH)**	25.46 (34.18)

### Internal consistency

The Amharic BPI responses showed good internal consistency with a Cronbach’s alpha value of 0.89 (alpha ranging from 0.84 to 0.88 if an item was deleted) for pain severity and Cronbach’s alpha 0.91 (0.89–0.92 if an item was deleted) for pain interference (See Table 3).

**Table 3:** BPI Internal consistency within items (2 factors)

Pain severity Items (α = 0.89)	Cronbach’s Alpha if Item Deleted	Pain interference Items (α = 0.91)	Cronbach’s Alpha if Item Deleted
Worst Pain	0.84	General Activity	0.89
Least Pain	0.86	Mood	0.90
Average Pain	0.85	Walking Ability	0.91
Pain Right Now	0.88	Normal Work	0.89
		Relationship with others	0.89
		Sleep	0.92
		Enjoyment Of Life	0.90

Good internal consistency was also observed in the 3-factor model, with α = 0.89 for pain severity, α = 0.84 for activity interference, and α = 0.86 for affective interference items (See Table 4).

**Table 4:** BPI Internal consistency within items (3 factors)

Pain severity Items (α = 0.89)	Cronbach’s Alpha if Item Deleted	Activity interference Items (α = 0.84)	Cronbach’s Alpha if Item Deleted	Affective Interference Items (α = 0.86)	Cronbach’s Alpha if Item Deleted
Worst Pain	0.84	General Activity	0.72	Mood	0.82
Least Pain	0.86	Walking Ability	0.83	Relationship with others	0.81
Average Pain	0.85	Normal Work	0.78	Sleep	0.87
Pain Right Now	0.88	–	–	Enjoyment Of Life	0.79

### Test-retest reliability

**Fig. 2:**
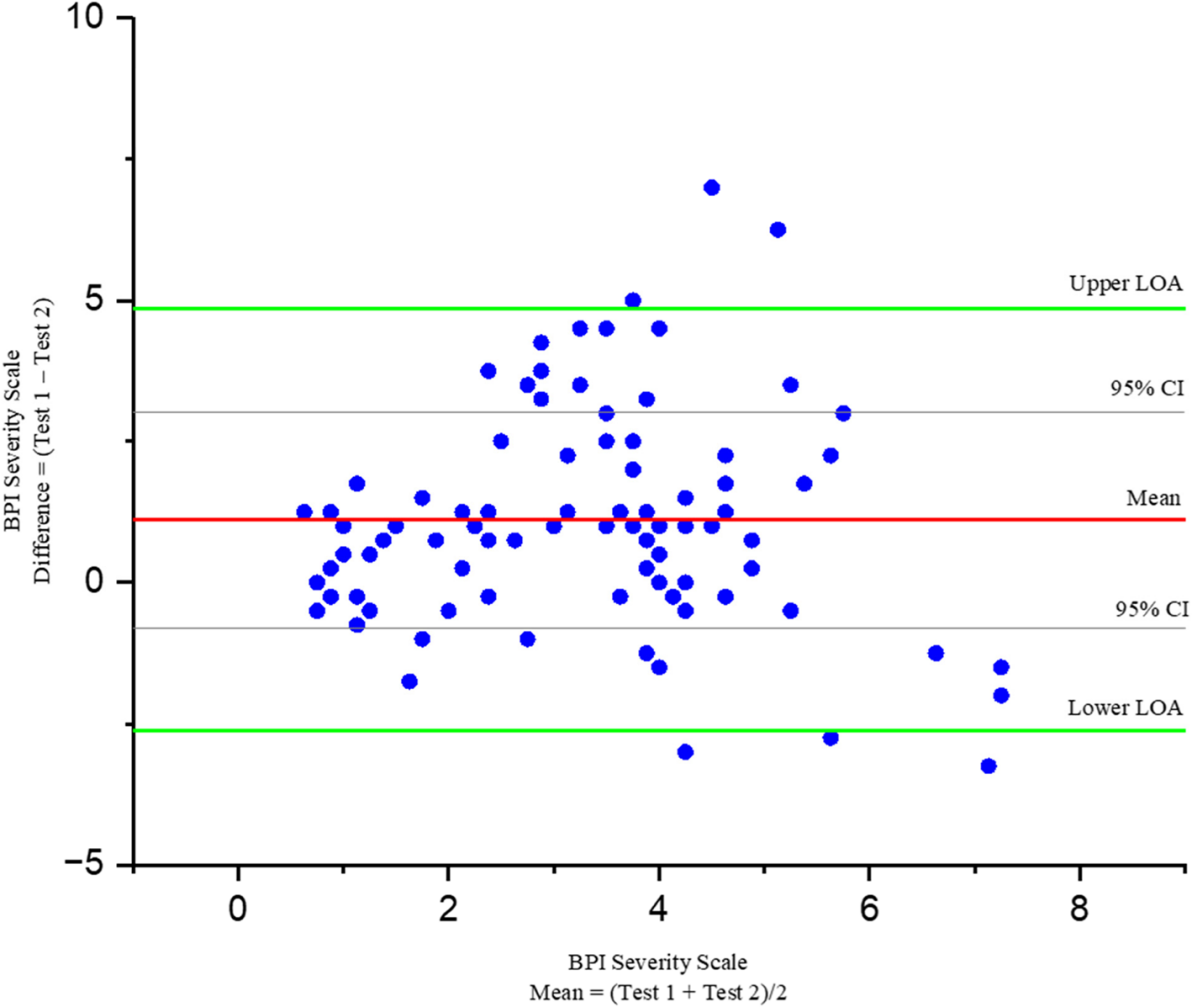
Bland-Altman plot for Amharic BPI pain severity scale. The Bland-Altman plot for the test-retest reliability of the Amharic BPI pain interference scale. The difference of Test 1 and Test 2 is plotted against their mean, together with the mean of the difference, 95% Confidence interval (CI), and upper and lower limit of agreement (LOI)

A total of ninety-one participants took part in the retest. The Amharic BPI was re-administered within three to 7 days of the initial assessment. The test-retest responses showed an intra-correlation coefficient (ICC) 0.82 (95% CI: 0.75–0.88) for the pain severity subscale and ICC 0.90 (95% CI: 0.87–0.93) for the pain interference subscale. The Bland-Altman plot for BPI pain severity scale showed the mean bias ±SD between the first assessment scores as 1.11 ± 1.91, and the upper and lower limits of agreement (LOA) were 4.85 and − 2.63, respectively (See Fig. 2).

**Fig. 3:**
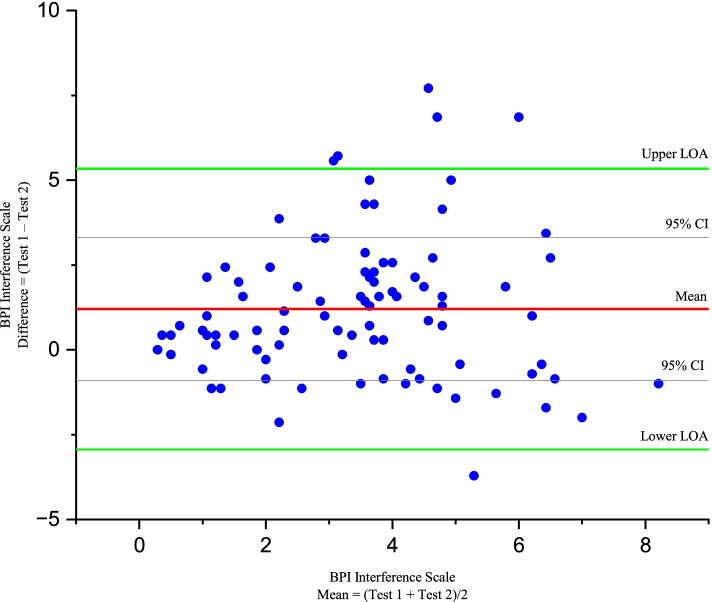
Bland-Altman plot for Amharic BPI interference scale

Similarly, the Bland-Altman plot for BPI pain interference scale showed the mean bias ±SD between the first assessment scores as 1.20 ± 2.11, and the upper and lower LOA were 5.34 and − 2.93 (See Fig. 3).

For both the scales, the majority of the scores are within the upper and lower level of agreement. To further inform us about potential proportional bias, we conducted a linear regression. The results suggested for BPI pain severity scale, an unstandardized beta (B) =0.02 and level of significance (α) =0.99, and for BPI pain interference scale, an unstandardized beta (B) =0.075 and level of significance (α) = 0.055 suggesting no significant proportional bias in test responses. The overall results indicate that the Amharic BPI pain severity and BPI pain interference responses demonstrate acceptable stability over time.

### Floor and ceiling effect

Three people (1.1%) and one person (0.4%) had the lowest score of 0 and the highest score of 9.5 on the pain severity scale, respectively. While one (0.4%) and one (0.4%) had the lowest score of 0 and the highest score of 9.5 on the pain interference scale, respectively.

### Convergent validity

Two hundred sixty-nine participants completed the Amharic BPI and the SF-36 Amharic questionnaire. As hypothesized, the BPI pain severity scale had the highest correlation with bodily pain scale of the SF-36 at r = − 0.44 (95%CI − 0.34 to − 0.53) and the BPI pain interference scale most closely correlated with physical functioning scale of SF-36 at r = − 0.63, (95%CI − 0.55 to − 0.70) (See Table 5).

**Table 5:** Convergent validity between the Amharic versions of BPI and SF-36 subscales (*N* = 269)

SF-36	BPI Mean Pain Severity (95% CI)	BPI Mean Pain Interference (95% CI)
General health perception (GH) Average	−0.43^a^ (−0.03 to −0.52)	−0.50^a^(−0.41 to −0.59)
Physical functioning (PF) Average	−0.41^a^(−0.30 to −0.50)	−0.63^a^(−0.55 to −0.70)
Role Functioning Physical (RLPH) Average	− 0.29^a^(− 0.18 to − 0.40)	− 0.48^a^(− 0.39 to − 0.56)
Role Functioning Emotional (RLEP) Average	−0.26^a^(− 0.15 to − 0.37)	−0.43^a^(− 0.33 to − 0.52)
Energy (EN) Average	−0.35^a^(− 0.24 to − 0.45)	−0.45^a^(− 0.35 to − 0.54)
Emotional Well-Being EM) Average	−0.33^a^(− 0.22 to − 0.43)	−0.41^a^(− 0.30 to − 0.50)
Social Functioning (SF) Average	−0.37^a^(− 0.265 to − 0.471)	−0.52^a^(− 0.43 to − 0.61)
Bodily Pain (BP) Average	−0.44^a^(− 0.34 to − 0.53)	−0.58^a^(− 0.50 to − 0.66)

Pearson correlation: ^a^Correlation is significant at the 0.01 level (1-tailed)

Sf = 36 scores: Higher scores indicate less impact on life, while lower scores indicate greater impact

BPI scores: Higher scores indicate a greater impact on life, while lower scores indicate a lesser impact

### Confirmatory factor analysis

**Fig. 4:**
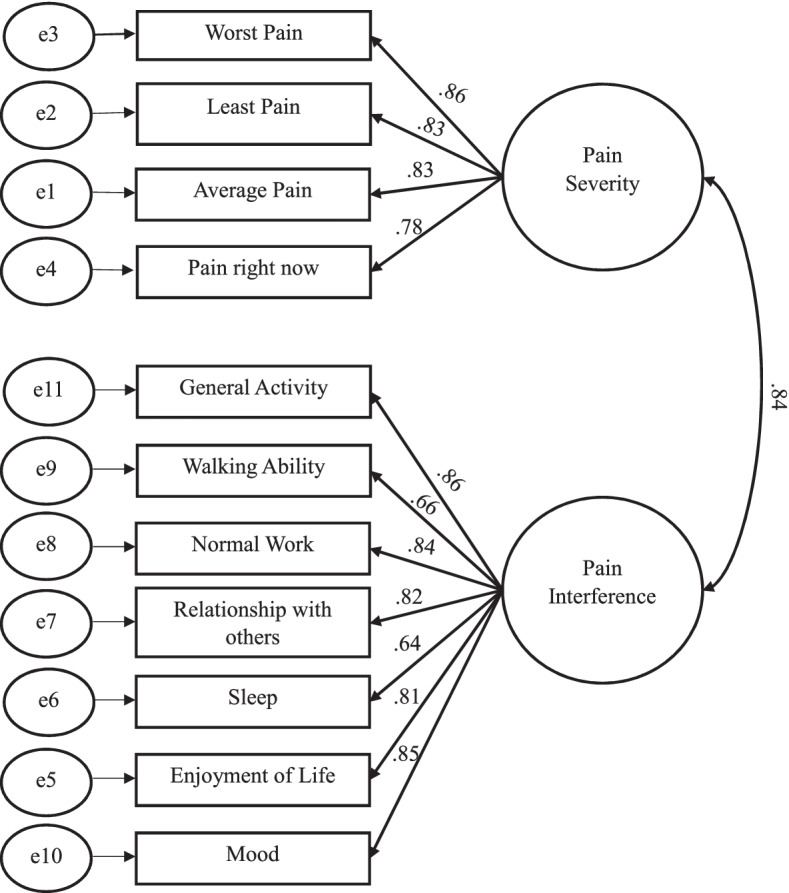
Confirmatory factor analysis for the 2-factor model (Standardized)

**Fig. 5:**
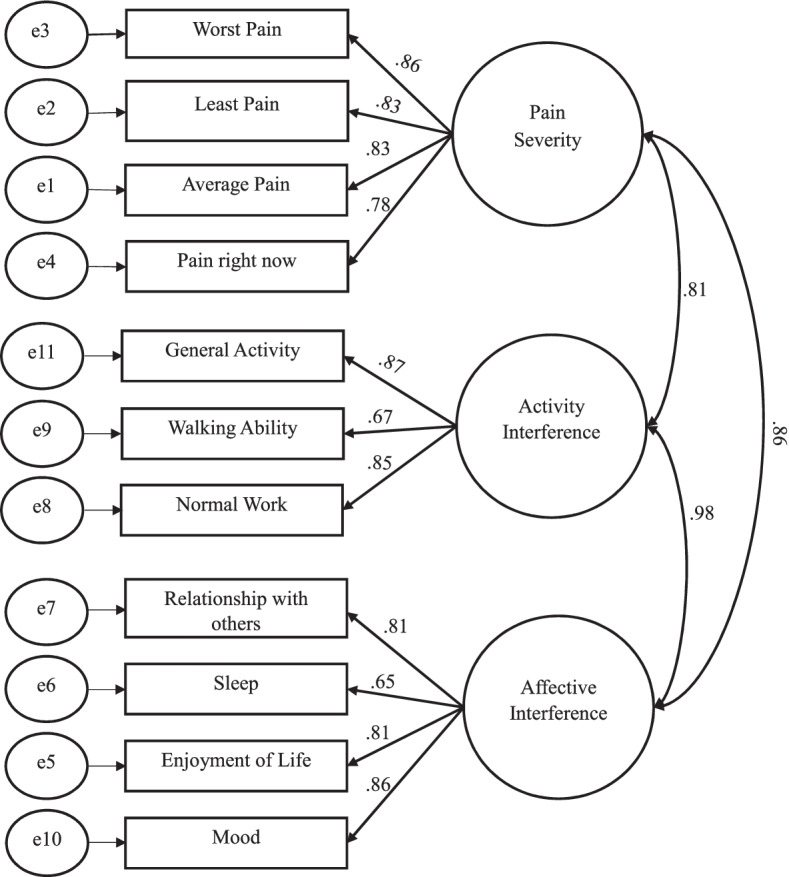
Confirmatory factor analysis for the 3-factor model (Standardized)

Confirmatory factor analysis demonstrated a comparable fit with the two-factor and three-factor models previously suggested [13, 16, 52]. For the two-factor model, results show a Chi-square = 125.368, degree of freedom = 43, Comparative Fit Index (CFI) = 0.962, and Tucker–Lewis’s index (TLI) = 0.951. For the three-factor model, results show a Chi-square = 120.534, degree of freedom = 41, Comparative Fit Index (CFI) = 0.963, and Tucker–Lewis’s index (TLI) = 0.950 (See Table 6). The standardized confirmatory models are also reported (see Fig. 4 and Fig. 5).

**Table 6:** Fit Indices for Confirmatory Factor Models

	Absolute/ predictive fit	Comparative fit		Parsimonious fit		Other fit Indexes				
Model	Chi-Square/ degree of freedom (X2/df)	Comparative Fit Index (CFI)	Tucker–Lewis’s index (TLI)	Parsimony Comparative Fit Index (PCFI)	Parsimony Goodness of Fit Index (PGFI)	Goodness of Fit Index (GFI)	Adjusted goodness of fit index. (AGFI)	Root mean square residual. (RMR)	Root mean Square Error of Approximation (RMSEA) (CI 90%)	Standardized Root Mean Residual (SRMR)
**2 Factors**	125.368/43 = 2.916	0.962	0.951	0.752	0.599	0.920	0.877	0.280	0.085(0.068–0.102)	0.0365
**3 Factors**	120.534/41 = 2.940	0.963	0.950	0.718	0.573	0.923	0.875	0.272	0.085(0.068–0.102)	0.0378

## Discussion

This study aimed to assess the validity and reliability of the Amharic BPI responses among patients with Chronic Primary Musculoskeletal Pain in Ethiopia and found good reliability and validity.

The Amharic BPI responses showed a good internal consistency of Cronbach’s alpha value of 0.89 for pain severity and Cronbach’s alpha 0.91 for pain interference which are above the acceptable cut-off points suggested in different studies, α ≥0.7 or α ≥0.8 [53, 54]. These results are also consistent with the alpha coefficient values estimated using the original tool (0.80–0.87 for severity and 0.89–0.92 for interference) and estimates of validity established using other translated versions of the BPI [10, 13, 16]. The results suggest that items within each scale are interrelated. The alpha coefficient values for both scales, “if an item was deleted,” were comparable to the overall alpha coefficient for the scales. This result suggests that each component within the scales contributes similarly related to the construct being measured (pain severity or pain interference).

The test-retest reliability assessment showed an ICC 0.82 (95% CI: 0.75–0.88) for pain severity suggesting “good” reliability and an ICC 0.90 (95% CI: 0.87–0.93) for the pain interference suggesting “good” to “excellent” reliability [55]. In comparison to the severity scale, the interference scale has higher test-retest reliability. This may be because the level of pain intensity is more likely to be variable over the three-to-seven-day interval between tests, while interference is more likely to be stable among patients with chronic pain during this interval [56]. Our findings were comparable with previous studies estimating the validity of BPI responses [10, 57]. Our study demonstrated a lower correlation coefficient than the BPI-Persian and BPI-German; however, these tests only included a 30–60 minutes test-retest interval time, which probably explains the higher correlation estimates in those studies [11, 16]. Our finding also included a Bland-Altman plot and the linear regression suggesting that there is no significant proportional bias between the two measurements and stability of the Amharic BPI responses over time.

The overall convergent validity test shows that the scores of the BPI-AM converge with that of the SF-36 scores. As expected, the BPI pain severity scale scores had a low to moderate correlation with the Bodily pain subscale of the SF-36 at r = − 0.44 (95%CI − 0.34 to − 0.53). A BPI-German validation study has also found a similar moderate correlation with the bodily pain subscale scores of the SF-36 [11]. A high correlation may not be expected given that the Bodily pain subscale score of the SF-36 is not a pure pain severity measuring item. Instead, it is an average of pain intensity and pain interference item. The Amharic BPI pain severity index had a low correlation with Role limitations due to physical health (r = − 0.29, 95%CI − 0.18 to − 0.40) and Role limitations due to emotional problems (r = − 0.26, 95%CI − 0.15 to − 0.37) items of the SF-36. The demographic characteristic of our population may explain these results. The majority of our participants were in the middle age group (mean age = 41.9), educated (92.6% had some form of education, worked (89.2% had some form of work), and had lived a long time with the pain (for 26 months on average). These demographic characteristics might contribute to the participant’s ability to overcome the role limitation due to physical health and emotional problems [58–60].

On the other hand, the BPI pain interference scale was moderately correlated with the SF-36 total score. This result shows that the BPI pain interference scale score correlates better with the overall SF-36 score than the BPI severity scale scores. These findings can be explained by the higher similarity in the SF-36 and BPI pain interference scale contents. The SF-36 is an eight-scale health-quality-of-life questionnaire that assesses two dimensions. These are the physical and mental dimensions [61]. Previous studies have also identified these dimensions for BPI pain interference scales [52, 62]. BPI pain interference scale moderately correlated with Physical functioning, Bodily pain, Social functioning, and General health scales of the SF-36. Radbruch et al. [11] also indicated similar correlations between BPI interference and SF-36 scores.

The BPI was developed to measure pain in two dimensions: pain severity and pain interference. The BPI interference scale was designed to capture two dimensions of interference: interference in activity and interference in affect [10]. Initial BPI tests determined two dimensions of the BPI, pain severity and interference [10, 11]. Meanwhile, other studies showed three dimensions: pain intensity, interference on activity, and affective interference [14, 52]. We assessed both suggested dimensions of BPI. All items loaded well in both models except the variable “Sleep”, which also loaded lower in previous models [10, 52]. Lower loading of “Sleep” might be due to the reason that there is no clear demarcation of “sleep” being grouped as “activity” or “affective” [10]. The X2/df, CFI, TLI, RMSEA, and SRMR values for the 2-factor and 3-factor Amharic BPI models found no significant difference to suggest one model over the other, and both models fit well. As a result, until further research examines the 3-dimensional structure, we recommend using the 2-dimensional structure of Amharic BPI, which is similar to the original BPI tool dimensions [10]. A systematic review finding from Jumbo et al. [63] on measurement property of BPI in pain related musculoskeletal conditions also strongly recommends the 2-dimensional structure of BPI.

The results show that Amharic BPI scores demonstrate good reliability and validity. Amharic BPI is the first assessment tool available to assess pain severity and pain interference in Ethiopia among patients with chronic primary musculoskeletal pain. This tool allows patients and health professionals to measure pain during an assessment, inform treatment, and monitor treatment outcomes. Self-reporting pain intensity and interference measures will also facilitate future clinical and epidemiological research on chronic primary musculoskeletal pain in Ethiopia.

### Limitations

There are some limitations identified in this study. During the data collection, participants were consistently provided with a paper copy of the Amharic BPI, and each item was read aloud to them. Participants provided their responses verbally. These data collection processes were chosen to mirror how the BPI would most often be used in clinical practice, given Ethiopia’s literacy rates [51]. However, this may limit the self-administration of the questionnaire, which may be more common in certain research contexts.

## Conclusions

Our findings showed that Amharic BPI scores demonstrate internal consistency, test-retest reliability, and construct validity among patients with chromic primary musculoskeletal pain in Ethiopia. Accordingly, the tool can be used in clinical practice or research in similar settings. Our findings support rating the Amharic BPI using the two-factor approach.

## Data Availability

This study is a part of Abey Abebe’s ongoing Ph.D. thesis. As a result, rather than putting the data in a public repository for the time being, we will share it upon reasonable request. The datasets used and/or analysed during the current study are available from the corresponding author upon reasonable request.

## Abbreviations

BPI: Brief Pain Inventory
ICC: Intraclass correlation coefficient
SF-36: Short Form Health Survey
SF-36-Am: Short Form Health Survey Amharic version
CFA: Confirmatory Factor Analysis
SPSS: Statistical Package for the Social Sciences
